# Anticancer Activity of Toxins from Bee and Snake Venom—An Overview on Ovarian Cancer

**DOI:** 10.3390/molecules23030692

**Published:** 2018-03-19

**Authors:** Marius Alexandru Moga, Oana Gabriela Dimienescu, Cristian Andrei Arvătescu, Petru Ifteni, Liana Pleş

**Affiliations:** 1 Department of Medical and Surgical Specialties, Faculty of Medicine, Transilvania University of Brasov, Brasov 500019, Romania; moga.og@gmail.com (M.A.M.); dimienescu.oana@gmail.com (O.G.D.); petru_ifteni@yahoo.com (P.I.); 2 Clinical Department of Obstetrics and Gynecology, The Carol Davila University of Medicine and Pharmacy, Bucharest 020021, Romania; lianamonicamaria@yahoo.com

**Keywords:** bee venom, snake venom, human cancer cell lines, ovarian cancer, carcinogenesis

## Abstract

Cancer represents the disease of the millennium, a major problem in public health. The proliferation of tumor cells, angiogenesis, and the relationship between the cancer cells and the components of the extracellular matrix are important in the events of carcinogenesis, and these pathways are being used as targets for new anticancer treatments. Various venoms and their toxins have shown possible anticancer effects on human cancer cell lines, providing new perspectives in drug development. In this review, we observed the effects of natural toxins from bee and snake venom and the mechanisms through which they can inhibit the growth and proliferation of cancer cells. We also researched how several types of natural molecules from venom can sensitize ovarian cancer cells to conventional chemotherapy, with many toxins being helpful for developing new anticancer drugs. This approach could improve the efficiency of standard therapies and could allow the administration of decreased doses of chemotherapy. Natural toxins from bee and snake venom could become potential candidates for the future treatment of different types of cancer. It is important to continue these studies concerning therapeutic drugs from natural resource and, more importantly, to investigate their mechanism of action on cancer cells.

## 1. Introduction

Cancer represents the disease of the millennium, a major problem in public health. Ovarian cancer is the fifth cause of death among the female population with an estimated 152,000 deaths worldwide and 239,000 new cases diagnosed annually, according to Reid et al. [1]. The American Cancer Society estimates that in the year 2018, 22,240 women will be diagnosed with ovarian cancer and 14,070 women will die from the disease in the USA [2,3]. Because of the undefined signs and symptoms, most patients are diagnosed in the advanced stages of the disease [4]. The management of ovarian cancer consists of initial surgery with staging [5], followed by chemotherapy and immunotherapy according to the disease stage [6]. 

Among the therapies used for ovarian cancer, chemotherapy remains the major option in cases where surgical treatment cannot be performed. The chemotherapeutic resistance or the incapacity of administrating chemotherapy because of the poor health status of the patient are important issues in these cases [7]. Development in the oncology field of new drugs from natural resources holds an important role in modern medicine, mostly because the standard treatments have serious side effects [8,9]. In some experimental studies, several plants and their compounds with possible anticancer effects have been reported [10,11,12], with their mechanisms being explained through the inhibition of angiogenesis, decreased cell growth and proliferation, apoptosis, and prevention of oxidation and inflammation [13,14]. However, compounds from plants are not enough nowadays. Therefore, in the last years, studies have focused on the anticarcinogenic effects of toxins from animal venom. Researchers have tried to develop different types of anticancer drugs from natural resources, considering them a new line of treatment, hoping that these substances could increase the efficiency of chemotherapeutic drugs [7,8,15].

## 2. Carcinogenesis Mechanism

Because of the need to develop new therapies that target genes or pathological pathways, various studies have been carried out to understand the events that transform a normal cell into a tumor cell [16]. As we described in our previous paper [17], carcinogenesis is a complex process that exerts several changes in a normal cell—namely initiation, promotion, and progression—and is a process that requires critical molecular and targeted pathways.

**Figure 1:**
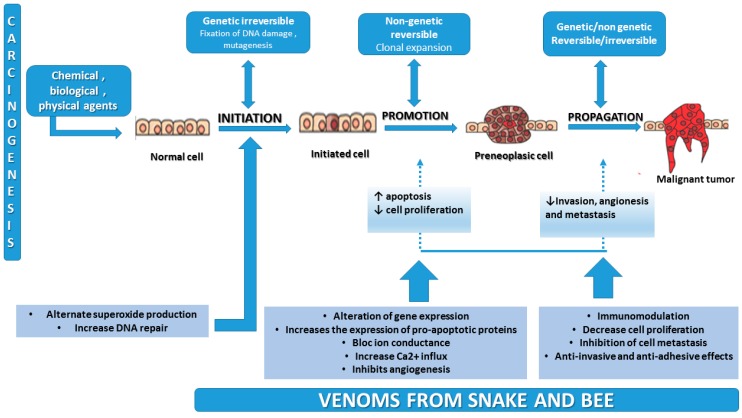
Carcinogenesis and the effects of toxins from snake and bee venom on the different steps of the process (adapted from [17,18]).

Figure 1 represents the process of carcinogenesis and shows the activity of natural toxins from venom that may block the main events of tumor formation.

## 3. Toxins from Animal Venom—Compounds and Activity

### 3.1. Bee Venom

Bee venom (BV) has been found to be useful in the treatment of different pathologies in traditional medicine: rheumatism, skin disease, arthritis, and malignant tumors [19]. Several studies have pointed out that bee venom stimulates an increased secretion of cortisol from the adrenal glands, a fact that can be observed as an anti-inflammatory effect, being used in the treatment of rheumatism and arthritis [20]. The anti-arthritis and anti-inflammatory activities (decreased expression of cyclooxygenase-2 and phospholipase A2 and decreased levels of interleukin-1, interleukin-6, tumor necrosis factor alpha, oxygen reactive species, and nitric oxide) have been reported in recent studies [19,20,21,22,23,24].

The effects of bee venom have been extensively studied by numerous researchers with recent studies pointing to several cytotoxic mechanisms such as apoptosis and necrosis, effects on growth inhibition and proliferation, and cytotoxicity and cell cycle alterations in various malignant cells. Several studies have reported the effects of natural toxins from bee venom on various types of cancers: breast [25], ovarian [26], bladder [27], lung [28], liver [29], and prostate cancer [30].

#### Compounds and Activity of Bee Venom

BV contains a variety of active compounds with various pharmaceutical activities, divided as follows: peptides including melittin, mast cell degranulating peptide (MCD), apamin and adolapin, enzymes (phospholipase A2), biogenic amines (histamine, dopamine, and norepinephrine), and non-peptic compounds (carbohydrates) [31]. Recent studies have investigated the effects of BV compounds, pointing out that some peptides (melittin, apamin, MCD, and adolapin) possess biological activity [32,33].

Melittin is the major component of bee venom, accounting for approximately 40–50% of dry weight. It is a protein that contains 26 amino acid residues. It is water soluble but also integrates and disrupts the phospholipid bilayers (natural or synthetic) [34,35]. Several reports have observed that melittin has various effects, including cell cycle arrest, apoptosis, and growth inhibition in different malignant cells, as well as antibacterial, anti-inflammatory, and antiviral properties [35,36,37,38].

Apamin contains 10 amino acids with two disulfide bonds and is the smallest neurotoxin in bee venom. The main effect is the selective inhibition of Ca^2+^-activated K^+^ channels [39]. Apamin blocks the channels at a minimum concentration, enhancing the synaptic plasticity and neuronal excitability. Ichii et al. reported the effect of apamin on tracheal contraction along with the release of histamine from lung tissues, pointing out that it decreases allergic airway inflammation [40]. A study from 2003 shows that apamin also inhibits nitric oxide-inducing relaxation of the myometrium in non-pregnant women [41]. 

Another important compound, MCD has 22 amino acid and includes two disulfide bonds between Cys5,19 and Cys3,15. The main effect is the inhibition of the release of histamine from mast cells at minimum concentrations with anti-allergic activity [42,43]. In the case of the inhibition of mast cell degranulation, studies suggest that this process is possible at concentrations higher than those that increase the release of histamine, mostly because it interacts with immunoglobulin E molecule [44]. 

Adolapin is another peptide from BV that possesses anti-inflammatory, antipyretic, and analgesic effects and inhibits the activity of phospholipase A2 [45]. The properties of this compound are due to the inhibition of the synthesis of prostaglandins through cyclooxygenase inhibitory properties [46]. 

Table 1 summarizes the major compounds of bee venom and their biological effects.

### 3.2. Snake Venom

Snake venom is synthesized from the venom glands of venomous snakes. It contains several different proteins, enzymes, peptides, and nucleotides [48,49]. It is well known that many snakes are harmless but some produce venom with different degrees of toxicity. The venom of every snake is different and researchers have observed that the venom differs among different species and according to the age of the snake and even their habitat or climate [50].

About 90–95% of the dry weight of snake venom consists of proteins that are responsible for the main biological effects. The venom has two main functions: (1) causes paralysis and (2) starts the digestive process. Hydrolysis of proteins and membrane components are due to the enzymes from the snake venom, the result being necrosis of the tissue and blood clotting. 

Venom is classified—according to its mode of action and its effects—into the following groups: cytotoxin, neurotoxin, cardiotoxin, and hemotoxin, containing several bioactive components that have various pharmacological activities [51]:

- Neurotoxins act at the level of the central nervous system, causing breathing difficulties or heart failure. These toxins affect the cholinergic neurons and block the binding of acetylcholine. Another effect is the inhibition of ion movement through the cell membrane, which blocks the communication between neurons [52].
- The toxins from snake venom that cause the destruction of the red blood cells are known as hemotoxins and mainly affect the circulatory system, blood function, and muscle tissue (causing gangrene and scarring). Viperidae species members (copperhead, rattlesnake, and cotton head) possess hemotoxic venom while mambas, cobras, krait, sea snakes, and coral snakes have neurotoxic venom. However, some species contain both neurotoxins and hemotoxins.
- The third group, the cardiotoxins, are those toxins that affect the heart muscle, binding to the cells of the heart and blocking muscle contraction [53].
- One of the most important and researched toxins from snake venom is the cytotoxin. It targets specific cellular sites, affecting the cell membrane or interfering with the transport of substances or the transduction of signals across the membranes [54].

Even if snake venom has important toxicological effects, new research regarding some of the venom compounds (proteins and peptides) points out that these substances could be used as pharmaceutical agents [9,55,56]. These compounds have proven antiviral effects against some types of viruses (yellow fever and dengue [57] and herpes simplex virus [58]), antimicrobial effects on Gram-positive and Gram-negative bacteria [59,60], antifungal activity [61], and antiparasitic effects on *Plasmodium falciparum* [62] and *Leishmania*.

#### Compounds and Activity of Snake Venom

Snake venom contains a mixture of different peptides, enzymes, proteins, chemicals, inorganic cations (sodium, zinc, calcium, magnesium, potassium), carbohydrates, free amino acids, and lipids [63]. At least 25 enzymes have been identified in different concentrations and combinations in snake venom [64].

Among the common enzymes identified are acetylcholinesterase, serine proteases, l-amino acid oxidase, phospholipases A(2) and metalloproteinases. We will discuss the most important enzymes from snake venom. 

Cholinesterase is the enzyme responsible for the effects on the central nervous system, having a major role in the cholinergic system where it is responsible for blocking nerve impulse transmission. It possesses high reactivity towards organophosphorus compounds. A study from 2011 pointed out that cholinesterase can be used as a treatment and prophylaxis of organophosphorus poisoning [65].

Being an endoglycosidase, hyaluronidase degrades the beta-*N*-acetyl-glucosaminidic linkages in HA polymers [66]. It is found in all snake venom and is known as a “spreading factor”, destroying the integrity of the extracellular matrix at the site of the bite and minimizing the local tissue destruction [67].

Phospholipase A2 can be found in high concentrations in snake venom. It is indispensable for various biological effects: cell growth and cell signaling, antiplatelet, anticoagulant, and hemolytic effects [68], as well as toxic effects such as neurotoxicity, cardiotoxicity, hypotensive, cytotoxicity, and proinflammatory [69,70]. Phospholipase A2 is composed of 120 amino acids and 14 cysteine residues that form seven disulfide bonds. It produces free fatty acids and lysophosphatidic lipid through catalyzing the calcium-dependent hydrolysis of the 2-acyl ester bond. It can also induce hydrolysis of phospholipid membranes, the consequence being the release of bioactive compounds [68]. It is categorized into two groups: 1PLA, identified mainly in the venom of cobras, sea snakes, and kraits and 2PLA from the venom of vipers and pit vipers [35,71,72]. 

l-amino acid oxidase (LAAO) represents 1–9%. It is a flavoprotein that catalyzes the stereospecific de-amination of l-amino acid substrate to an alpha-keto acid, producing ammonia and hydrogen peroxide [73]. It has been observed that LAAO from snake venom has an affinity for hydrophobic amino acids and through the generation of high levels of hydrogen peroxide may induce apoptosis in endothelial cells [73]. 

Table 2 summarizes the main compounds of snake venom and their activity. 

## 4. Effects of Bee and Snake Venom on Cancer Cells

### 4.1. The Effects of Bee Venom on Cancer Cells

Bee venom acts through several mechanisms to induce apoptosis of malignant cells and inhibit tumor growth. The activation of phospholipase A2 by the main compound of BV, melittin, is the most important mechanism [47]. Gajski et al. observed that melittin has an inhibitory effect on calmodulin as an antiproliferation agent of bee venom and contributes to the increased PLA2 activity, calcium influx and necrosis [76]. 

Several studies have reported that melittin causes hemolysis by disrupting erythrocyte membranes. It also exerts a cytotoxic effect on malignant cells by inhibiting tumor growth and inducing the activation of matrix metalloproteinases (MMP) and caspase, which are responsible for apoptosis and necrosis [77,78,79]. The lytic activity is low when melittin is associated with target peptides (immunoconjugate of melittin, melittin–avidin conjugate, adenovirus–melittin and RGD–melittin conjugate) [78,80,81]. Other researchers have reported that the conjugation of melittin with gene therapy and hormone receptors can be considered as a new target therapy for different types of cancer, such as breast and prostate cancer, but extended research is needed [82,83]. A study from 2011 has reported on the activities of bee venom compounds regarding their cancer mechanisms, concluding that venom can inhibit prostate cancer by inactivating NF-κB and in this way alters the caspase pathway [30]. The report by Holle et al. [78] used a melittin–avidin conjugate, pointing out that this association has a strong cytotoxic activity on prostate malignant cells. They investigated the cytolytic effects against normal cells in vitro, concluding that the conjugate had decreased cytotoxic activity against normal L-cells. When tumors were injected in vivo with the melittin–avidin conjugate, the tumor size decreased compared to non-injected tumors. 

Ip et al. studied the activity of BV on different cancer cells, concluding that in the case of human breast cancer (MCF7 cells), the bee venom compounds can induce apoptosis by activating caspase-9 and -3 or through the release of EndoG and AIF from mitochondria [25]. The authors also investigated the mechanism through which bee venom induces apoptosis in human bladder cancer TSGH-8301 cells. They reported multiple pathways: inducing the release of reactive oxygen species and Ca^2+^ and ER stress-mediated apoptotic death, and promoting the activation of the initiation of caspases and effector caspase with adaptor proteins (Fas/CD95), and acting as a receptor for BV [25]. Regarding lung cancer, a study from 2010 reported that the compounds of bee venom have anti-angiogenic effects through blocking tyrosine phosphorylation of VEGFR-2 [28]. The pharmacological activity of melittin was also evaluated in leukemic U937 cells. BV produces downregulation of ERK and Akt signal pathways with Bcl-2 and caspase-3 as the key regulators, inducing apoptosis [79]. 

### 4.2. The Effects of Snake Venom on Cancer Cells 

The cytotoxicity of various compounds from snake venom is explained by the alterations in the cellular metabolism that leads to several effects on cancerous cells [8]. According to these observations, many researchers have tried to develop several chemotherapeutic drugs based on the results of the cytotoxic ability of the toxins produced by animals. The first report was conducted by DeWys et al. who observed that the defibrination process resulted after the administration of Ancrod (a polypeptide from *Agkistrodon rhodostoma*) and, followed by cyclophosphamide, decreases the tumor weight and activates fibrinolysis. In the same report, other mechanisms such as platelet aggregation were observed to be involved in the decrease in the tumor dimensions [84]. A study in vivo concluded that the venom of *Naja nigricollis* inhibited, through these mechanisms, the growth of melanoma [85]. 

Another researcher studied the inhibitory effects of this venom on tumors in vivo and in vitro, with a possible application in cancer therapy. Song et al. concluded that this activity was proven by the expression of pro-apoptotic proteins such as caspase-3 and Bax, which increased while the levels of Bcl-2 (an anti-apoptotic protein) decreased [86]. 

In the last decades, studies have been carried out to point out the antitumoral potential of peptides (cytotoxins and cardiotoxins) from different species of snakes. 

The first studies regarding the effects of snake venom on sarcoma cells were performed by Braganca et al. [87,88]. The researchers investigated the effects of the venom from *Naja naja* snake on sarcoma cell cultures, calling it cobra venom factor (CVF). The mechanism through which cardiotoxin-3 (CTX-3) from *Naja naja atra* venom exercises its effects on tumors was studied by Yang et al. [89] who reported that apoptosis is followed by increased expression of Bax and endonuclease G and decreased expression of Bcl-x in K562 cells. Another report showed that CTX-3 possesses apoptotic effects through the activation of the JNK pathway and caspase-12 by triggering Ca^2+^ influx, the consequence being the rapid increase in the cytosolic Ca^2+^ concentration [90].

Chien et al. reported in two studies on the antiproliferative effects of CTX-3 on HL-60 leukemia cells. They concluded that CTX-3 induces apoptosis by activating the c-JUN-*N*-terminal kinase and increasing the sub-G1 population, and by activating the mitochondrial apoptosis pathway and endoplasmic reticulum pathway, resulting in an increased level of related protein 78 (GRP 78) and Ca^2+^ [91,92].

Several investigations were conducted on human breast cancer cells too, more exactly, on MDA-MB-231 cells [93] and MCF-7 cells [94]. In the first case, apoptosis was confirmed by the loss of mitochondrial membrane potential and accumulation of the sub-G1 population, while in the second cell type it was observed that CTX-3 suppressed the proliferation and induced apoptosis by downregulating NF-kB in the cells. 

The toxins from snake venom also showed activity on metastasis [95]. The integrins, being an important cell surface receptor, are demonstrated to be involved in cell–cell and cell–matrix interactions. 

Disintegrins are found in snake venom and are an inhibitor of integrin-dependent cell adhesion and platelet aggregation [96,97]. Hong et al. in 2003 described a disintegrin purified from the venom of a Korean snake, salmosin. Disintegrins induce apoptosis by competing with the extracellular matrix through direct binding to integrin [98]. Contortrostatin (CN) is another disintegrin that has been purified from the venom of the southern copperhead snake. CN has high affinity interactions with different integrins from cancerous cells and vascular endothelial cells, resulting in antitumor activity. A study from 2004 described a more relevant delivery system for CN, the liposomal CN (LCN), and concluded that this antitumor agent accumulates at the tumor site where it exercises its action on tumor growth and angiogenesis and curtails tumor metastasis [99]. The antimetastatic activity of CTX III isolated from *Naja naja atra* [100] was investigated by Lin et al. The downregulation of the activity and expression of matrix metalloproteinase MMP-9 was observed. This effect was caused by the inactivation of PI3K/Akt signaling pathways and p38 MAPK and NF-κB activity. This activity inhibits the migration and invasion of cells that cause breast cancer. 

Cytotoxins from *Naja* species of snakes possess activity against the A549 cells (human lung adenocarcinoma) and HL 60 cells (promyelocytic leukemia); more exactly CT1 and CT2 from *Naja oxiana*, CT1 from *Naja haje*, and CT3 from *Naja kaouthia* [101]. Vierira Santos et al. also observed in their study on Ehrlich ascites tumor (EAT) growth that *Bothrops jararaca* venom (BjV) induces an increase in mononuclear leukocytes and inhibits EAT growth [102]. 

Among other toxins from the snake venom from the Viperidae and Crotilidae families, metalloproteinases (SVMPs) are major components with different biological properties. The effects of these toxins vary from inhibition of platelet aggregation, coagulation factor activation, and fibrinolytic activities to possible anticancer properties such as apoptotic and proinflammatory activities [103]. A study from 2014 [104] pointed out that cancer cell adhesion is interrupted by Jararhagin, a purified snake venom metalloproteinase from *Bothrops jararaca*. The authors concluded that the potential effect on melanoma cells is exerted through the increased antiproliferative and caspase-3 activities. Wan et al. [105] also investigated metalloproteinases from snake venom and identified a basic SVMP from *Trimeresurus stejnegeri* venom that induces morphological modifications and inhibits the proliferation of ECV304 cancer cells.

Another major compound of snake venom that has the potential to inhibit cancer cells is the lectins (polyvalent carbohydrate-binding proteins). Pereira–Bittencourt et al. [106] showed an inhibitory effect of BJcuL (lectin isolated from *Bothrops jararacussu* snake venom) on eight cancer cell lines of which CFPAC-1 (pancreatic cancer cell line), Caki-1, and A-498 (renal cancer cell lines) showed the most promising results with an inhibitory concentration of 50%. A study from 2001 [107] pointed out the cytotoxic effects of BJcuL in MKN45 and AGS cells (gastric cancer cell lines), through altering cell adhesion and inducing apoptosis. In the same study, the authors investigated lebecetin, a C-type lectin from *Macrovipera lebetina* venom. The results showed that this lectin has anti-integrin activity, being able to inhibit the adhesion, migration, and invasion of the tumor cells [35].

## 5. Studies Regarding the Effects of Toxins from Bee and Snake Venom on Ovarian Cancer Cells

In the case of ovarian cancer, surgery is the main therapy depending on the staging [4], followed by chemotherapy, which is used for the purpose of removing the residual cancer cells. Among the chemotherapeutic drugs used for the management of ovarian carcinoma are cisplatin, paclitaxel, and carboplatin; however, many patients develop chemoresistance [18,108]. Several studies were conducted during the last years to improve the treatment modalities for ovarian cancer, especially with natural toxins that can be added to the therapeutic drugs in order to increase the response to therapy. Researchers have investigated the effects of bee venom components on ovarian cancer cells, pointing out the activity of toxins from the venom on this type of cancerous cell [109]. 

### 5.1. Bee Venom and Ovarian Carcinoma

Holle et al. designed an MMP2 cleavable melittin–avidin conjugate, the study being based on the affirmation that melittin administered alone is very toxic for cells, inducing cell lysis, but in association with avidin it becomes inactive. They observed with in vitro studies that this conjugate exerts a high cytolytic effect on ovarian cancer cells (SKOV-3), cells that possess a strong MMP2 activity, and decreased activity on normal L-cells that possess low MMP2 activity. In vivo studies showed decreased tumor dimensions of the ones injected with melittin–avidin conjugate, concluding that through the cytolytic activity and tumor targeting ability, the conjugate melittin–avidin can be used in the treatment of ovarian carcinoma and is being considered as a promising approach for cancer therapy [78].

Another study from 2007 [110] pointed out the effect of melittin on ovarian cancer by describing that in vivo the ovarian cancer tumors were decreased in the group treated with melittin and in vitro they observed that melittin inhibits the growth and proliferation of ovarian cancer cells.

The mechanism through which melittin and other bee compounds can inhibit the ovarian cancer cells were described by Jo et al. in 2012 [26]. The authors investigated the pathways of inhibition of ovarian cell growth when bee venom and melittin were used. They concluded in their report that bee venom at a dose of 1–5 μg/mL and melittin (0.5–2 μg/mL) can induce apoptosis in the SKOV-3 and PA-1 ovarian cancer cells, depending on the administered dose. The mechanism of action on carcinogenesis is linked to the expression of death receptor 3 and 6 that were found to be increased in both ovarian cellular cancer lines and DR 4 that was found in an increased level only in the PA-1 cells. After the treatment with melittin and bee venom, the phosphorylation of JAK2 and STAT3 and the expression of Bcl-2 was inhibited, while the expression of caspase-3, caspase-8, and Bax was increased. 

Liu et al. [29] generated a fusion protein that can inhibit tumor growth in vivo since cytokines, such as IL-2, are very important in the immune response in the case of cancer cells. They observed that melittin increases the immune function by enhancing Th1 cells function and chose to develop a fusion protein (melittin–MhIL-2) consisting of a mutant hIL-2 genetically linked to melittin. This fusion protein exerts activities of both IL-2 and melittin, thereby inhibiting the growth and proliferation of the ovarian cancer cells SKOV-3 in vitro and in vivo. This makes the fusion protein melittin–MhIL-2 a potential anticancer agent.

Because more patients are becoming chemoresistant to the usual chemotherapeutic drugs, several studies have been conducted to investigate the synergistic effects of bee venom in combination with cisplatin on ovarian cancer cells. In 2012, Alizedehnohi et al. [111] evaluated the cytotoxic effects of bee venom alone and in combination with cisplatin on A2780cp cells, cisplatin-resistant ovarian cancer cells. The treatment with 8 µg/mL bee venom or 25 mg/mL cisplatin for 24 h resulted in almost 50% cisplatin-resistant A2780cp cell death. Similar results were observed in the simultaneous treatment with bee venom at 4 µg/mL and cisplatin at 10 mg/mL for 24 h, concluding dose-dependent effects. The authors also investigated the effects on the expression of Bcl-2, the results showing that the expression of Bcl-2 in A2780cp cells decreased when the cells were treated with bee venom and cisplatin. The conclusions of the study were that bee venom has an effect on human ovarian cancer cells and an enhanced cytotoxic effect on the antitumor agent cisplatin. Another study from 2015 [112] investigated the potential cytotoxic and pro-apoptotic effects of bee venom and chrysin (natural flavonoid derived from honey and propolis) on A2780cp cisplatin-resistant human ovarian cancer cells. Their results pointed out that bee venom 8 μg/mL, chrysin 40 µg/mL and 6 + 15 μg/mL bee venom + chrysin resulted in approximately 50% cell death in A2780cp cells compared with the control group. This study is concordant with the one of Alizedehnohi et al. [111] regarding the downregulation of Bcl-2. They concluded that the mechanism through which bee venom and chrysin decreased the expression of ovarian cancer cells are the following: ROS accumulation, inhibition of Bcl-2, and caspase activation via a mitochondrial pathway. The increased expression of caspase-3 and caspase-9 and the downregulation of Bcl-2 indicate that this type of treatment has antitumor activity through the intrinsic apoptotic pathway. New research needs to be done in this field since bee venom can improve ovarian cancer therapy and also the platinum agent resistance, the result being the possible decrease of the mortality in this type of cancer.

In Table 3 the studies regarding the effects of bee venom compounds on different ovarian cancer cells are exemplified.

### 5.2. Snake Venom and Ovarian Carcinoma

We identified two studies related to the activity on ovarian cancer cells of contortrostatin (CN), a disintegrin from snake venom. In 2001, Markland et al. [113] investigated the effects of CN on OVCAR-5 (human epithelial carcinoma cell line of the ovary). They observed that this disintegrin inhibits tumor invasion and blocks the adhesion of OVCAR-5 to extracellular matrix proteins [103]. Another study by Swenson et al. [114] observed the anti-angiogenic and antitumor effects of contortrostatin from the venom of *Agkistrodon contortrix contortrix.* The authors used human ovarian cancer cells (A2780) injected intraperitoneally into 40 female Athymic nude mice. They concluded after examination that the group treated with CN showed a dramatic decrease in the numbers and size of the tumors formed. The authors also developed an effective method of delivery with less adverse effects—the liposomal encapsulation of CN (LCN)—that possesses a high efficiency in inhibiting tumor dissemination and angiogenesis in human ovarian cancer cell line following intravenous administration.

Another disintegrin recently investigated is saxatilin from *Gloydius saxatilis* [115]. The observations from the report of Kim et al. [116] showed that another type of ovarian cancer cell line named MDAH 2774 was inhibited under the effects of TNF-α and decreased MMP-9 mRNA expression.

De Carvalho et al. [117] investigated the effect of BJcuL, an important lectin from snake venom, on human ovarian cancer cells (OVCAR-5). They observed a weak adherence of the cancer cells to BJcuL. They could not demonstrate the inhibition of adhesion to the extracellular matrix proteins of lectin but concluded that the viability of the tumor cells was suppressed by BJcuL and, therefore, concluded that the lectin BJcuL can inhibit the proliferation and growth of tumor cells and endothelial cells. 

Another mechanism of snake venom is the programmed cell death of ovarian cancer cells by inhibiting the translocation of p65 and p50 and inhibiting NF-kB and STAT3 signaling. This pathway was observed in the case of toxin from *Vipera lebentina turnica.* The authors observed that the toxin upregulated the expression of caspase-3 and Bax and decreased the expression of Bcl-2 anti-apoptotic protein [86].

Table 4 shows the studies regarding the effects of snake venom on the ovarian cancer cell.

## 6. Conclusions and Future Perspectives

Ovarian cancer is the fifth neoplasm among women worldwide, especially in developing countries such as Romania where the annual mortality rate has increased with an average of 1%/year since 1990. It represents a major problem for public health mainly because of the undefined signs and symptoms that are an impediment to early diagnosis and treatment. 

Various toxins from venom have shown cytotoxic effects on human ovarian cancer cell lines, providing new perspectives in drug development. Natural toxins from animal venom are bioactive compounds that have been demonstrated to have possible anticarcinogenic properties. The potential therapeutic uses of animal toxins have received great interest from researchers and are currently in the early stages of observation. Several studies have been published that have evaluated the involvement of snake and bee venom on specific points of carcinogenesis. The proliferation of tumor cells, angiogenesis, and the relationship between cancer cells and the components of the extracellular matrix are important in the events that occur in carcinogenesis; these pathways are being used as targets for new anticancer treatments. 

In this review, we identified the effects of toxins from bee and snake venom, mostly the anticarcinogenic activity in several types of cancer, focusing on ovarian cancer. The anticarcinogenic activity of animal venom depends on the origin of the cancer line. Nowadays, knowledge about the cytotoxic mechanism of venoms is still not fully known. Only a few in vivo and in vitro studies focusing on the anticarcinogenic effects of snake and bee venom on ovarian cancer and how they can contribute to the development of new drugs have been conducted.

We pointed out that natural toxins from bee and snake venom hold potential in the therapy of ovarian cancer because they interfere in carcinogenesis by modulating the critical processes of cellular proliferation, differentiation, apoptosis, angiogenesis, and metastasis. Specifically, these toxins inhibit the proliferation and growth of ovarian cancer cells by inducing apoptosis and growth arrest, by interacting with integrins via glycoprotein receptors located on cellular surfaces, and by modulating the signal transduction pathways. 

Another important problem in the management of ovarian cancer is the resistance to chemotherapy. In the case of ovarian cancer, chemotherapy is an important tool in the treatment. Chemotherapy increases patient survival rates and destroys cancerous cells, but the main issue is that these agents also destroy other dividing cells such as hematopoietic stem cells and epithelial cells. In this paper, we pointed out that various compounds from bee and snake venoms can sensitize ovarian cancer cells to conventional chemotherapy, with the target tumor toxins being helpful for developing novel anticancer therapeutics. This combined approach could improve the efficiency of standard therapies and allow decreases in the doses of chemotherapy drugs, leading to reduced adverse side effects.

An important challenge is to integrate the new molecular findings into clinical practice and to identify the major venom components and their specific targets and to investigate them in clinical trials. With the advancements made in the field of molecular biology, it is now possible to produce recombinant toxins and to use them to design new drugs. Studies that focus on the natural toxins from animal venom should continue to provide researchers with an improved understanding of carcinogenesis and anticancer mechanisms. 

In conclusion, the studies from our review indicate that several toxins from bee and snake venom could become potential candidates for the future treatment of ovarian cancer. We summarized some of the bee and snake bioactive compounds that have been studied to date for their possible anticancer therapeutic properties. It is important to continue searching for therapeutic drugs from natural resources, as well as investigate their mechanism of action in cancer cells.

## Figures and Tables

**Table 1:** Bee venom components and their biological effects (adapted from Son [19] and Osrolic [47]).

	Compound	Biological Effects
**Peptides**	Melittin	anti-inflammatory, immunostimulatory, immunosuppressive, antibacterial, antifungal, antiviral, cytotoxic effect, ↑ the activity of phospholipase A2, anti-atherosclerotic, endosomolytic, stimulates smooth muscles, activates the hypophysis and adrenal glands, ↑ capillary permeability by ↑ blood circulation and ↓ blood pressure, ↓ blood coagulation
	Apamin	cytotoxic effect, anti-inflammatory, anti-serotonin action, immunosuppressive, activates the hypophysis and adrenal glands, nociceptive effect
	MCD peptide	lyses mast cells, releases histamine, serotonin and heparin, ↑ capillary permeability, anti-inflammatory, analgesic effect, simulates the central nervous system
	Adolapine	inhibits PLA2 activity, inhibits COX activity, ↓ inflammation and ↓ pain, antipyretic, inhibits the aggregation of erythrocytes
	Protease inhibitor	inhibits the activity of trypsin, chymotrypsin, plasmin, thrombin, ↓ inflammation, anti-rheumatic
	Procamine A, B, Secapine, Panime, Minimine, Tertiapine, Cardiopep, Melittin F	
**Proteins** **(Enzymes)**	Phospholipase A2	immunomodulatory, anti-inflammatory, destroys phospholipids and dissolves the cell membrane of blood bodies; ↓ the blood coagulation and blood pressure, prevents neuronal cell death caused by prion peptides, cytotoxic effects against cancer cells, antitumor effects
	Phospholipase B	detoxicating activity
	Hyaluronidase	catalyzes the hydrolysis of proteins, dilates blood vessels and ↑ permeability, causing an ↑ in blood circulation; immune response, tissue spread activity
	Glucosidase, Acid phosphomonoesterase	
**Biogenic Amines**	Histamines	dilates blood vessels, ↑ the permeability of blood capillaries and ↑ blood circulation; stimulates smooth muscles
	Dopamine, Norepinephrine	
**Others**	Carbohydrates, r-Aminobutyric acid, B-aminoisobutyric acid	

**Table 2:** Snake venom components and properties (adapted from Koh [74] and Fatima [75]).

**Proteins**	**Enzymes**	**Compound**	**Major Activity**
		Acetylcholinesterase	Paralysis
		Bglucosaminidase	Tissue damage
		Phosphoesterase	Anticoagulant, paralysis
		Phospholipase A2	Membrane damage, anticoagulant
		Hyaluronidase	ECM damage, apoptosis
		l-amino acid oxidase	Platelet effect, anticoagulant
		Snake venom metalloproteases	Anticoagulant, cell damage
		Snake venom serine proteases	Anticoagulant, fibrinogenemia
	**Non-Enzymes**	Protein C activator	Anticoagulant
		Growth factors (INGF, VEGF)	Endothelial damage, edema
		Inhibitor of the prothrombinase complex formation	Anticoagulant
		Lectins	Platelet effect
		Precursors of bioactive peptides	Smooth muscle inhibitor
		Von Willebrand factor binding proteins	Anticoagulant
		Cysteine-rich secretory proteins	Anticoagulant
	**Peptides**	Cytotoxic, cardiotoxic, myotoxic, neurotoxic	Anticoagulant, inflammatory
		Disintegrins	Apoptosis, myotoxicity
		Natriuretic	Platelet effect, vascular
		Bradykinin potentiator	Hypotensive
**Organic Compounds**	Biogenic amines	Serotonin, histamine	
	Amino acids, carbohydrates, citrate, nucleosides		
**Inorganic Compounds**	Calcium, cobalt, copper, iron, phosphorus, potassium, magnesium, sodium, zinc		

**Table 3:** Studies of the effects of bee venom and their components on ovarian cancer cells.

Study	Compound	Mechanism	Cancer Cell	Results
Alizadehnohi et al. [111]	Melittin	induces apoptosis in cisplatin-resistant ovarian cancer cells	A2780CP	cell death and cytotoxic effect, cells exposed entered an early stage of apoptosis simultaneous treatment with both BV and cisplatin ~50% A2780cp cell death; Bcl2 expression was markedly decreased compared to the control group
Alonezi et al. [118]	Melittin	compared the effects of melittin in combination with cisplatin	A2780 (cisplatin-sensitive) A2780CR (resistant ovarian cancer cells)	reduction of metabolites in the TCA cycle, oxidative phosphorylation, purine and pyrimidine metabolism, and the arginine/proline pathway. melittin-cisplatin combination—stronger effect on the A2780 cell line compared to the A2780CR cell line
Amini et al. [112]	Bee venom and chrysin	cytotoxic and pro-apoptotic effects of BV and chrysin	A2780CP (cisplatin- resistant human ovarian cancer cells)	co-treatment induced 50% cell death in A2780cp cells compared with controls, showed down-regulation of Bcl-2; ROS generation and apoptotic cell death with exposure to BV or chrysin or BV + chrysin co-treatment. BV and chrysin triggered apoptosis through the intrinsic pathway
Holle et al. [78]	Melittin/avidin conjugate	cytotoxic effects	SK-OV-3	activity higher in SK-OV-3 compared to L-cells; melittin/avidin conjugate lysed SK-OV-3 cells induced cell lysis in cultured cells, dependent on MMP2 activity (since significant MMP2 activity is observed only in SK-OV-3); cell death was observed in SK-OV-3 cells; decreased tumor size in vivo.
Jo et al. [26]	Melittin	inhibits cell growth through enhancement of DR expressions	SKOV3 PA-1	induced programmed cell death; ↑ expression of DR 6 and DR3 in both cancer cells, but expression of DR4 ↑ only in PA-1 cells ↑expression of death receptors pro-apoptotic proteins (Bax, caspase-3, and caspase-8) inhibited the phosphorylation of STAT3 and JAK2 and also the expression of Bcl-2; cleaved caspase-3 was ↑ in SKOV3 while cleaved caspase-8 was ↑ in PA-1 cells
Lee et al. [119]	Melittin	suppresses the proliferation and growth of tumor cells	SKOV3 PA-1	induced programmed cell death; expression of DR6 and DR3 ↑ in both cancer cell lines, expression of DR4 ↑ only in PA-1 cells inhibited the STAT3 pathway
Liu et al. [29]	Melittin-MhIL-2 fusion protein	inhibits cell growth and proliferation of ovarian carcinoma	SKOV3	directly inhibited the growth of human ovarian cancer cells in vitro; inhibited tumor growth in human ovarian cancer cells in mice model and exhibited enhanced antitumor activity compared to rhIL-2
Su et al. [120]	Recombinant human Upa1-43-melittin	inhibits growth of ovarian cancer cells	SKOV3	suppressed growth of SKOV3 induced cell cycle arrest and induced SKOV3 cells apoptosis fusion protein does not have any obvious toxicity on normal tissues
Su et al. [121]	ATF-melittin	cytolytic activity	SKOV3	rATF-melittin inhibited the proliferation and growth of SKOV3 cells no cytotoxicity effect on normal cells
Xu et al. [110]	Melittin	inhibits the growth and activity of proliferation of ovarian cancer	SKOV3	the average weight of ovarian cancer in the melittin group was lower than that of the control group. in vitro melittin inhibited the growth of SKOV3 cells

**Table 4:** Studies of effects of snake venom and their components on ovarian cancer cells.

Study	Compound	Species	Mechanism	Cancer Cell	Results
Markland et al. [113]	Contortrostatin	*Agkistrodon contortrix*	inhibits tumor cell invasion and adhesion	OVCAR-5	inhibited ovarian cancer dissemination; inhibited angiogenesis inhibited cancer cell line adhesion and invasion CN blocked the invasion of cancer cells through the inhibition of the vß5 function
Swenson et al. [114]	Contortrostatin	*Agkistrodon contortrix*	CN has cytotoxic and anti-angiogenic activity in human ovarian cancer animal model	A2780 SEAP	inhibited A2780 SEAP tumor formation inhibited tumor burden inhibited cancer cell proliferation and angiogenesis
Lipps et al. [122]	Atroporin and Kaotree	*Crotalus atrox* *Naja naja kaouthia*	Atroporin has higher cytolytic activity on SKOV-3 than the compound Kaotree	SKOV-3 HBT 77	the combination of the two compounds showed elevated cytotoxic activity on the ovarian cancer cells
Kim et al. [116]	Saxatilin	NR	decreased cell invasion through the regulation of MMP-9 activity in MDAH 2774 inhibits tumor progression	MDAH 2774	regulated integrin-mediated signaling reduced cell migration by physically blocking integrin. levels of MMP-9 mRNA decreased after saxatilin treatment; bFGF or actin levels were unchanged TNF-α-induced MMP-9 activities were suppressed by saxatilin treatment
Carvalho et al. [117]	BJcuL	*Bothrops jararacussu*	BJcuL binds the tumor cells but does not inhibit adhesion of these cells to fibrobronectin, laminin, and type I collagen. BJcuL does not interfere with ECM protein-binding cell surface receptors such as integrins.	OVCAR-5	Ovarian cells adhered to BJcuL but significantly weaker when compared to fibronectin; BJcuL was ineffective in blocking adhesion of OVCAR-5 to fibronectin, laminin, and type I collagen. when the cell lines OVCAR-5 were exposed to BJcuL for 96 h, a cytotoxic effect of this lectin could be seen BJcuL had different effects on the viability of tumor cells, depending on its concentration; cytotoxic to the cells at concentrations higher than 1 mM. Using OVCAR-5 cells, the effect of FBS in the medium on BJcuL cytotoxicity was clearly demonstrated BJcuL exerted a higher cytotoxic effect on the cells suspended in medium containing 5% FBS than on those suspended in medium containing 2.5% FBS.
Song et al. [86]	NR	*Vipera lebetina turanica*	induces programmed cell death inhibits the proliferation and growth of ovarian cancer	PA-1 SK-OV3	In SKOV-3 human ovarian cancer cells the inhibition of growth and proliferation was observed ↑ the expression of the Bax and caspase-3 pro-apoptotic proteins and ↓ the expression of Bcl-2 anti-apoptotic protein In the control group not treated with toxin an increased DNA binding activity of NF-κB was observed In the group treated with snake venom, the inhibition of the translocation of p65 and p50 and an inhibition of DNA binding activity of STAT3 was observed

## Abbreviations

The following abbreviations are used in the manuscript:

AIF	apoptosis-inducing factor
Akt	protein kinase B
ASR	age standardized rate
Bax	BCL2-associated X protein
Bcl-2	B-cell lymphoma 2
bFGF	basic fibroblast growth factor
BJcuL	lectin from the venom of the snake *Bothrops jararacussu*
BjV	*Bothrops jararaca* venom
BV	bee venom
CN	contortrostatin
COX	cyclooxygenase
CTX-3	cardiotoxin-3
CVF	cobra venom factor
DR	death receptor
DU 145	a prostate cancer cell line
EAT	Ehrlich ascites tumor
ECM	extra cellular matrix
EndoG	endonuclease G
ER	endoplasmic reticulum
ERK	extracellular signal-regulated kinase
GRP 78	protein 78
hIL-2	human interleukin-2
IL-2	interleukin-2
JAK2	Janus-associated kinase 2
JNK	c-Jun *N*-terminal kinases
LAAO	l-amino acid oxidase
LCN	liposomal encapsulation of contortrostatin
MAPK	mitogen-activated protein kinase
MCD	mast-cell degranulating peptide
MCF-7	human breast adenocarcinoma cell line
MCF7- cells	human breast adenocarcinoma cell line
MhIL-2	melittin human interleukine-2
MMP	matrix metalloproteinases
NF-κB	nuclear factor-kappa B
NR	not reported
OVCAR 5	human epithelial carcinoma cell line of the ovary
PLA2	phospholipase A2
RGD	arginyl-glycyl-aspartic acid
rhIl-2	recombinant human IL-2 protein
STAT 3	signal transducer and activator of transcription 3
TCA	tricarboxylic acid
Th1	lymphocyte T helper 1
TNF-α	tumor necrosis factor-α
VEGF	vascular endothelial growth factor
VEGFR	vascular endothelial growth factor receptor
